# Annular pancreas in Trichorhinophalangeal syndrome type II with 8q23.3-q24.12 interstitial deletion

**DOI:** 10.1186/s13039-015-0201-0

**Published:** 2015-12-15

**Authors:** Qi Li, Zhen Zhang, Yuchun Yan, Ping Xiao, Zhijie Gao, Wei Cheng, Lin Su, Kaihui Yu, Hua Xie, Xiaoli Chen, Qian Jiang, Long Li

**Affiliations:** Department of Pediatric Surgery, Capital Institute of Pediatrics, No. 2 Yabao Rd, Beijing, 100020 China; 2Department of Radiology, Capital Institute of Pediatrics Affiliated Children’s Hospital, Beijing, China; Department of Pathology, Capital Institute of Pediatrics Affiliated Children’s Hospital, Beijing, China; Department of Neurology, Capital Institute of Pediatrics Affiliated Children’s Hospital, Beijing, China; Department of Surgery, Beijing United Family Hospital, Beijing, China; Department of Paediatrics and Surgery, Faculty of Medicine, Nursing and Health Sciences, Monash University, Victoria, Australia; Reproductive Medicine Center, Clinical College of PLA Affiliated Anhui Medical University, Hefei, China; Department of Pathophysiology, School of Preclinical Sciences, Guangxi Medical University, Nanning, China; Municipal Key Laboratory of Child Development and Nutriomics, Capital Institute of Pediatrics, Beijing, China; Department of Medical Genetics, Municipal Key Laboratory of Child Development and Nutriomics, Capital Institute of Pediatrics, Beijing, China

**Keywords:** Annular pancreas, 8q23.3-q24.12 deletion, Trichorhinophalangeal syndrome type II, Chinese, qPCR

## Abstract

**Background:**

Trichorhinophalangeal syndrome type II (TRPS II, OMIM # 150230) is a rare autosomal dominant genetic disorder characterized by craniofacial and skeletal abnormalities. Loss of functional copies of the *TRPS1* gene at 8q23.3 and the *EXT1* gene at 8q24.11 are considered to be responsible for the syndrome.

**Case Presentation:**

Herewith, we report an 8-year-old girl with sparse scalp hair, bulbous nose, thin upper lip, broad eyebrows, phalangeal abnormalities of both hands/toes, multiple exostoses, mild intellectual impairment and severe malnutrition. In addition, the patient also had annular pancreas, a rare co-existing feature in patients with TRPS II.

**Conclusions:**

A contiguous 5.47 Mb deletion involving 8q23.3-q24.12 was detected by array comparative genomic hybridization (aCGH), leading to haploinsufficiency of 10 protein coding genes, 1 long non-coding RNA and 1 microRNA. Quantitative PCR (qPCR) examination confirmed half-reduced DNA copy of the patient and normal expression of both parents, indicating a *de novo* origin of the deletion and complete penetrance of the mutation.

## Background

Trichorhinophalangeal syndrome type II (TRPS II), also known as Langer–Giedion syndrome (LGS), is a contiguous gene deletion syndrome involving loss of functional copies of multiple genes on 8q24.1. Hall et al. first described this disorder in 1974 [1], which combines the clinical features of trichorhinophalangeal syndrome type I (OMIM # 190350) and multiple exostoses type I (OMIM # 133700). Affected individuals are characterized by multiple dysmorphic facial features including large, laterally protruding ears, a bulbous nose, elongated upper lip, sparse scalp hair, winged scapulae, multiple cartilaginous exostoses, and mental retardation. Although familial cases have been occasionally observed [2], most reported cases to date are sporadic ones. In 1984, Buhler and colleagues identified an interstitial deletion involving chromosome 8q24 in an individual with TRPS II [3], and later on proposed this complex disease as a chromosome deletion syndrome involving both *TRPS1* and the gene for multiple hereditary exostoses *EXT1*. Patients with TRPS II often show clinical variability depending on the loss of additional genes in the deleted region [4]. For example, mild to moderate intellectual disability, conductive hearing loss, epilepsy, congenital nephrotic syndrome, growth hormone deficiency, and a submucosal cleft palate have all been described as additional features of TRPS2/LGS depending on the deletion size [5–9].

Here, we present an 8-year-old female with sparse scalp hair, facial dysmorphism, malaligned crowded teeth, multiple exostoses, winged scapulae, annular pancreas, mild intellectual impairment and an 8q23.3-q24.12 deletion detected by aCGH.

## Case presentation

**Fig. 1:**
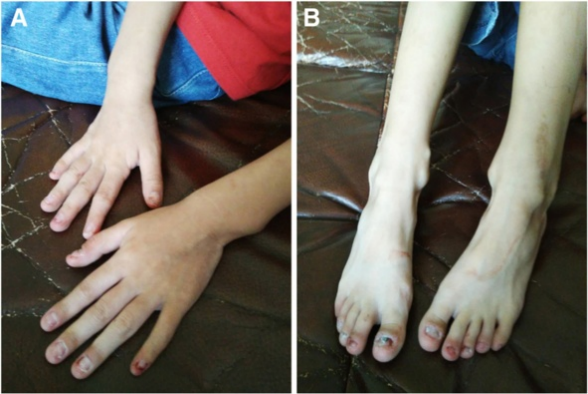
Clinical features of our patient diagnosed with trichorhinophalangeal syndrome type II: **a**, long and clubbed-fingers. **a**, **b** Nails of fingers and toes are typically thin and dystrophic

**Fig. 2:**
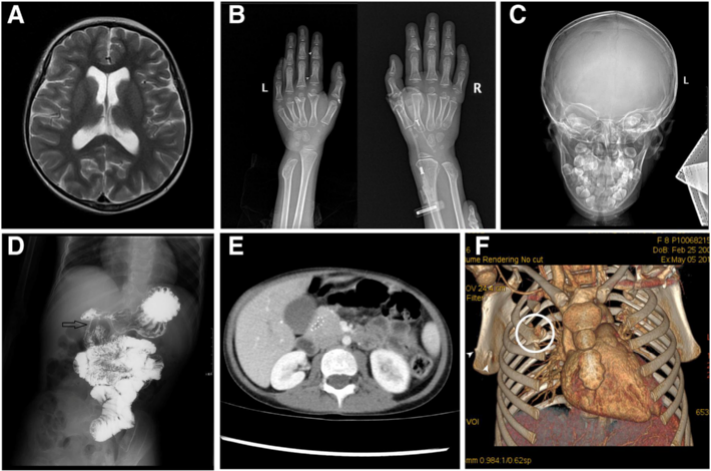
Imaging examinations of the patient. **a** Brain MRI showed encephalatrophy-like changes. **b**, **c** X-ray examination showed short metacarpals of the fifth finger, cone-shaped epiphyses of the proximal inter-phalangeal joint of middle phalanx of the fingers (white arrow), multiple exostoses (white arrow head) and malaligned crowded teeth. **d** Upper GI series showed insufficient distention at the pylorus and duodenum (black arrow) indicating encircled pancreatic tissue. **e** Axial CT image clearly showed annular pancreatic tissue encircling the second portion of the duodenum (white circle). **f** CT volume rendering revealed exostoses (white arrow head) and fusion malformation of the second and third ribs on the right side (white circle)

Our female patient was the first child of healthy unrelated parents of normal intelligence. She was born by caesarian section at 40 weeks of gestation after an uneventful pregnancy. Her birth weight was 3050 g (<50^th^ centile), length 49 cm (<50^th^ centile) and head circumference 33 cm (<50^th^ centile). Both parents were normal, and there was no family history of congenital malformations. The patient was referred to our Department of Pediatric Surgery at 8 years of age with recurrent vomiting and severe malnutrition. On clinical examination, the girl had brachycephaly, fine and sparse hair, a bulbous tip of the nose, long and flat philtrum, thin upper lip, broad eyebrows, malaligned crowded teeth, micrognathia, microcephaly (with a head circumference of 42 cm at the age of 8-year-and-5-month), hypotonia and hyper-extensibility of the joints. In addition, she had winged scapulae, long and clubbed-fingers. Her finger and toe nails were typically thin and dystrophic (Fig. 1). Her weight was only 13.25 kg (<1^st^ centile), and her height was 120 cm (<15^th^ centile). She showed mild intellectual impairment and psychomotor retardation. Heart and abdominal ultrasound revealed no pathological abnormalities. Brain MRI showed encephalatrophy-like changes (Fig. 2, a). X-ray examination showed short metacarpals of the fifth finger, cone-shaped epiphyses of the proximal inter-phalangeal joint of middle phalanx of the fingers and malaligned crowded teeth (Fig. 2, b and c). Upper GI (gastrointestinal) series showed insufficient distention at the pylorus and duodenum indicating encircled pancreatic tissue. Axial CT image clearly showed annular pancreatic tissue encircling the second portion of the duodenum (Fig. 2, d and e). CT volume revealed exostoses of the scapulae and fusion malformation of the second and third ribs on the right side (Fig. 2, f). Informed consent and permission of publication of identifiable photographs of the patient have been obtained.

## Results

**Fig. 3:**
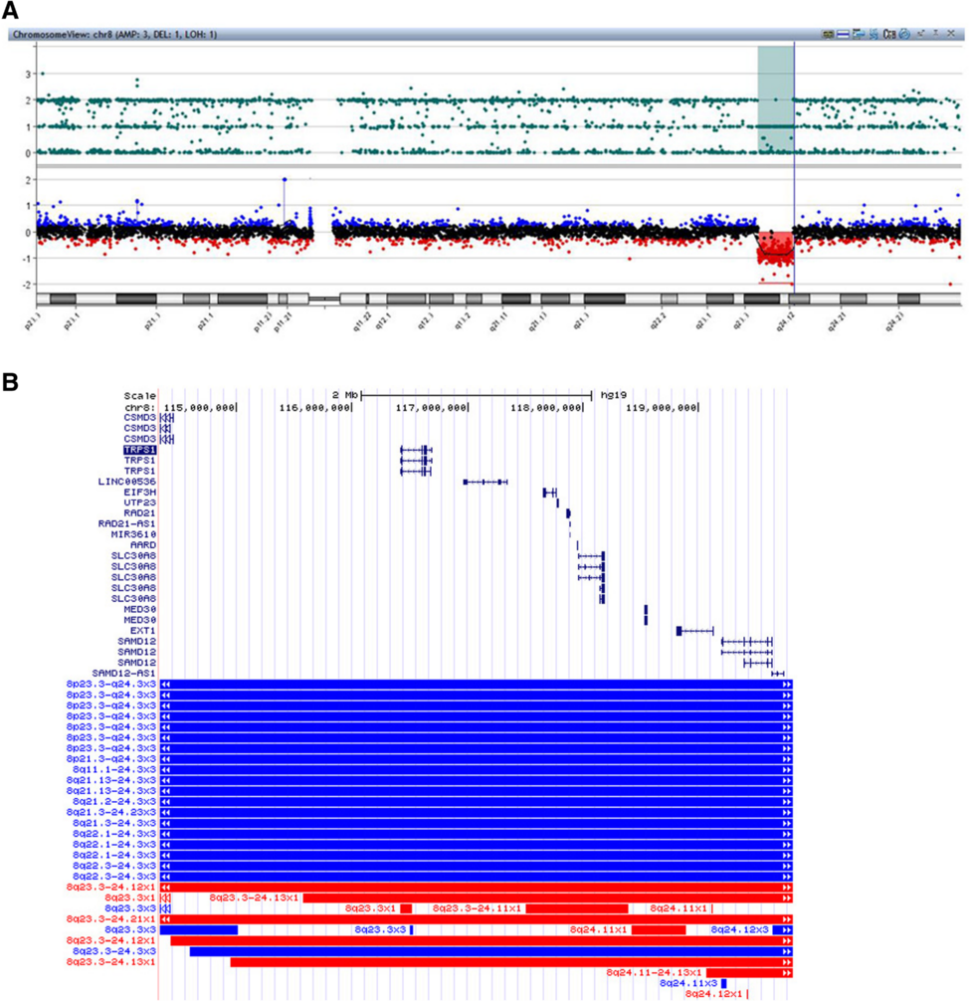
aCGH on the patient blood shows a 5.47 Mb deletion at chromosome bands 8q23.3-q24.12 (114,338,391-119,817,276, hg19). (**a**) Chromosomal view and (**b**) UCSC zoom in view

**Fig. 4:**
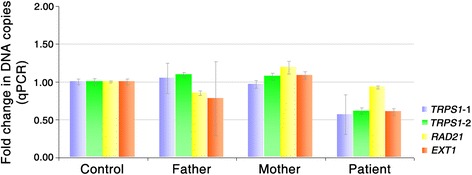
qPCR examinations show approximately half-reduced DNA copy of the patient and normal expression of both parents, indicating a *de novo* origin of the deletion

Cytogenetic analysis at a resolution of 400 bands displayed a normal female karyotype (46, XX). Whole-genome aCGH analysis on the patient detected a 5.47 Mb deletion at chromosome bands 8q23.3–q24.12 (114,338,391–119,817,276, hg19). The deleted region encompasses altogether 10 protein coding genes (*CSMD3*, *TRPS1*, *EIF3H*, *UTP2*3, *RAD21*, *AARD*, *SLC30A8*, *MED30*, *EXT1* and *SAMD12*), 1 long non-coding RNA (*LINC00536*) and 1 microRNA (*MIR3610*) (Fig. 3). Targeted gene sequencing revealed no pathological mutation of *TRPS1* and *EXT1* gene in the patient (data now shown). Quantitative PCR tests show approximately half-reduced DNA copy of the patient and normal expression of both parents, indicating a *de novo* origin of the deletion (Fig. 4).

## Discussion

Here we report the identification of a novel deletion of 8q23.3–q24.12 responsible for a Chinese patient suffering from Trichorhinophalangeal syndrome type II. Quantitative PCR confirmed the heterozygous variant which leads to haploinsufficiency of 10 protein coding genes, 1 long non-coding RNA and 1 microRNA. Both parents show a normal expression level as control, indicating a *de novo* origin of the deletion and complete penetrance of the mutation.

The trichorhinophalangeal syndrome (TRPS) is a rare autosomal dominant disorder characterized by craniofacial dysmorphism and bone deformities. Depending on the severity of the phenotype, three subtypes have been described: TRPS I (OMIM # 190350) and TRPS III (OMIM # 190351) are caused by mutations in *TRPS1*; and TRPS II (OMIM # 150230) is caused by a contiguous gene deletion affecting *TRPS1* and *EXT1* amongst others. Diagnosis of TRPS is normally based on clinical features and radiographic examination [10]; however, genetic analysis is often helpful especially in the case of non-classical clinical presentation [11, 12]. To date, more than 100 patients with TRPS have been reported in the literature [5], and features common to all three subtypes include sparse, slowly growing scalp hair, laterally sparse eyebrows, pear-shaped nose and large, protruding ears. Further typical characteristics include a long flat philtrum and a thin upper lip. In addition, short hands (feet) and impaired growth are common in all types of TRPS, while body weight is usually normal in relation to height. With respect to the head circumference, it is typically normal throughout life, except for those with a genomic deletion in whom one-third could have head circumference below the 3^rd^ percentile [13].

The most distinguishable feature for TRPS II from type I and III is exostoses, which are only present in those individuals with a deletion that extends to *EXT1*. Furthermore, intellectual disability (developmental delay) is present in most but not all TRPS II patients, typically mild to moderate in severity, and does not seem to correlate with the size of the deleted segment. Maas et al. summarized the information on 103 cytogenetically or molecularly confirmed TRPS individuals, and suggested that the size of contiguous gene deletions vary considerably in TRPS II and there are no common breakpoints [5, 11]. In our patient, aCGH analysis revealed the deletion of both *TRPS1* and *RAD21* genes which is in accordance with the dysmorphic facial features. Multiple exostoses, winged scapulae, impaired growth and mild intellectual impairment highly support the clinical diagnosis of TRPS II. Interestingly, the patient was also found to have annular pancreas, which, to our knowledge, is a novel co-existing feature in patients with TRPS II. Annular pancreas is a relatively rare congenital anomaly [14] characterized by an extension of pancreatic tissue around the second part of the duodenum and is thought to represent aberration in the development of the ventral pancreatic bud [15]. Recent studies highlight the role of the hedgehog signaling pathway in the development of this anomaly [16]. Overexpression of the ventral-specific gene transmembrane 4 superfamily member 3 (*tm4sf3*) has also been associated with annular formation [17]. Moreover, isolated case reports of familial annular pancreas have also been documented, suggesting a genetic basis for the development of this anomaly [18]. The 8q deletion in our patient spans 5.47 Mb and none of the genes within this region seemed to be related to the hedgehog signaling pathway. Further study is warranted to provide an answer to the underlying pathological mechanism.

## Conclusions

In summary, we present molecular cytogenetic characterization of a *de novo* deletion involving 8q23.3–q24.12 in an 8-year-old female with clinical features of TRPS II and annular pancreas. This is the first molecularly diagnosed patient with typical TRPS II in China. Our findings further confirm the relationship between TRPS II and 8q24.1, and expand the phenotypic and molecular spectrum of the disorder.

## Materials and methods

### array comparative genomic hybridization (aCGH)

DNA from peripheral blood was isolated by the Blood and Tissue kit (Qiagen, Valencia, CA). Whole-genome aCGH on patient’s blood was performed using Agilent CNV + SNP 180 K oligonucleotide array (Agilent Technologies Inc., Palo Alto, CA). This array has 170,334 probes and the overall median probe resolution is 11–13 kb.

### Quantitative polymerase chain reaction (qPCR)

Quantitative polymerase chain reaction (qPCR) was carried out in the presence of SYBR Green measuring the fluorescence signal produced by the binding of SYBR Green to the studied amplicons and compared with a reference sample. Average copy numbers of *GAPDH* in normal candidates (copy numbers = 2) were used as endogenous control. Real-time qPCR was performed with 7500 Fast Real-Time PCR System (Life, USA). The copy numbers of *TRPS1*, *RAD21* and *EXT1* were calculated by using the comparative C (T) method. The qPCR program was as follows: start at +50 °C for 2 min, then at +95 °C for 10 min, followed by 40 two-step cycles (15 s at +95 °C, 1 min at +60 °C). Reactions were adjusted to a final volume of 20 ul/well using 2 ul of genomic DNA (10 ng/ul), 1 ul of both of primers (10 pmol/ul), 6 ul of sterile water and 10 ul of Master Mix. The primers for *GAPDH* are (forward) 5’-AATGGGCAGCCGTTAGGAAA-3’ and (reverse) 5’-AAAAGCATCACCCGGAGGAG-3’. The primers for target genes are as following: *TRPS1*-1 (forward) 5’-AAGCACTCTAAAGCCACATGC-3’ and (reverse) 5’-TGTGCCAAGAACTTTTCCAG-3’; *TRPS1*-2 (forward) 5’-AATGCAAATGGCGGATATGT-3’ and (reverse) 5’-CCAGGCCAACACTGCTTTAT-3’; *RAD21* (forward) 5’-TGGGCCTGATAGTCCTGATT-3’ and (reverse) 5’-GCTCCAATGCAAATGCTTCT-3’; *EXT1* (forward) 5’-TGTGACAAAATTGCCTCCAA-3’ and (reverse) 5’-GCTGAACCACCAGTGAGTGA-3’. Statistical analysis was performed using Fisher’s exact test. *P* < 0.05 was considered statistically significant.

## Consent

Written consent was obtained from the patient and her parents for publication of this Case report and any accompanying images. A copy of the written consent is available for review by the Editor-in-Chief of this journal.

## Abbreviations

aCGH: Array comparative genomic hybridization
LGS: Langer–Giedion syndrome
qPCR: Quantitative polymerase chain reaction
TRPS II: Trichorhinophalangeal syndrome type II
